# Differences in bacterial taxa between treatment-naive patients with major depressive disorder and non-affected controls may be related to a proinflammatory profile

**DOI:** 10.1186/s12888-024-05547-z

**Published:** 2024-01-31

**Authors:** Julie Kristine Knudsen, Caspar Bundgaard-Nielsen, Peter Leutscher, Simon Hjerrild, René Ernst Nielsen, Suzette Sørensen

**Affiliations:** 1 Centre for Clinical Research, North Denmark Regional Hospital, Bispensgade 37, Hjørring, 9800 Denmark; 2 Department of Clinical Medicine, Aalborg University, Aalborg, Denmark; 3 Steno Diabetes Center North Denmark, Aalborg, Denmark; 4 Psychosis Research Unit, Aarhus University Hospital, Aarhus, Denmark; 5 Department of Clinical Medicine, Aarhus University, Aarhus, Denmark; 6 Department of Psychiatry, Aalborg University Hospital, Aalborg, Denmark

**Keywords:** Microbiota, Microbiome, Gut-Brain Axis, Depression, Major depressive disorder

## Abstract

**Background:**

Major depressive disorder (MDD) is characterized by sadness and anhedonia, but also physical symptoms such as changes in appetite and weight. Gut microbiota has been hypothesized to be involved in MDD through gut-brain axis signaling. Moreover, antidepressants display antibacterial properties in the gastrointestinal tract. The aim of this study was to compare the gut microbiota and systemic inflammatory profile of young patients with MDD before and after initiation of antidepressant treatment and/or psychotherapy in comparison with a non-depressed control group (nonMDD).

**Methods:**

Fecal and blood samples were collected at baseline and at follow-up after four and twelve weeks, respectively. Patients started treatment immediately after collection of the baseline samples. The gut microbiota was characterized by 16 S rRNA gene sequencing targeting the hypervariable V4 region. Plasma levels of 49 unique immune markers were assessed using Mesoscale.

**Results:**

In total, 27 MDD patients and 32 nonMDD controls were included in the study. The gut microbiota in the baseline samples of MDD versus nonMDD participants did not differ regarding α- or β-diversity. However, there was a higher relative abundance of the genera *Ruminococcus* gnavus group, and a lower relative abundance of the genera *Desulfovibrio*, *Tyzzerella, Megamonas, Olsenella, Gordonibacter, Allisonella* and *Rothia* in the MDD group compared to the nonMDD group. In the MDD group, there was an increase in the genera *Rothia*, *Desulfovibrio, Gordinobacteer* and *Lactobacillus*, while genera belonging to the Firmicutes phylum were found depleted at twelve weeks follow-up compared to baseline. In the MDD group, IL-7, IL-8 and IL-17b levels were elevated compared to the nonMDD group at baseline. Furthermore, MDI score in the MDD group was found to correlate with Bray-Curtis dissimilarity at baseline, and several inflammatory markers at both baseline and after initiation of antidepressant treatment.

**Conclusion:**

Several bacterial taxa differed between the MDD group and the nonMDD group at baseline and changed in relative abundance during antidepressant treatment and/or psychotherapy. The MDD group was furthermore found to have a pro-inflammatory profile compared to the nonMDD group at baseline. Further studies are required to investigate the gut microbiota and pro-inflammatory profile of patients with MDD.

**Supplementary Information:**

The online version contains supplementary material available at 10.1186/s12888-024-05547-z.

## Introduction

Major depressive disorder (MDD) has a lifetime prevalence spanning between 2% and 20% depending on socioeconomic and cultural characteristics in addition to the methods of characterization employed in each individual study [1]. Patients with a history of MDD have increased morbidity, mortality and a lower quality of life compared to age-matched controls, such as an overall relative risk of dying at 1.81 compared to non-depressed controls, and a 9.3 − 23% higher likelihood of having comorbid diseases [2, 3]. Furthermore, the total economic burden of the disorder in Europe alone accounts for 1.1 billion euro in annual costs [4]. The etiology of MDD is multi-factorial and includes genetic and environmental factors, such as a family history of mood disorders, being female or if the patient has experienced sexual or childhood trauma or have lived in a negative environment [5–7]. The two core symptoms associated with depression are depressed mood and anhedonia [8], but several somatic features are common in patients with depression. These include changes in appetite or abdominal symptoms such as pain and bloating [9], as well as a pro-inflammatory profile [10]. The amount and severity of non-mental symptoms have been positively associated with worse mental symptoms and poorer treatment outcomes [9] and especially inflammatory markers have been predicted to correlate to response to SSRIs [11]. Moreover, research has suggested a causal link between MDD and the gut microbiota, as fecal microbiota transplantation from patients with MDD into recipient animals have induced depressive-like behavior [12–16].

Several of the bacteria inhabiting the gut sends signals from the gastrointestinal system to the brain through the gut-brain axis [17]. This can happen through stimulation of the enteric nervous system [18], or by production of metabolites which can penetrate the blood-brain barrier [19]. For example, short chain-fatty acids (SCFAs) have been found to produce several beneficial health features, such as decreased permeability across the blood-brain barrier, which are proposed to function through signaling in the gut-brain axis [20–23]. On the other hand, cell wall components of gram-negative bacteria such as lipopolysaccharide (LPS), have been observed to induce inflammatory responses in human peripheral blood mononuclear cells [24]. Additionally, intestinal bacteria produce serotonin, a neurotransmitter, by stimulating secretion from intestinal enterochromaffin cells [25]. The intestinal commensals exist in a symbiotic relationship with the human host, as they provide us with essential vitamins, such as vitamin B and K, and amino acids [26–28], drive the maturation of the immune system [29, 30], and protect us from invading pathogens [31, 32]. Several studies have assessed the gut microbiota in patients with MDD [12, 13, 33, 34], and while diversity indices and several bacterial taxa were found to be significantly different between patients with MDD and non-affected controls, very few of them agree on which bacterial species were significant, as well as whether they were less or more prevalent in patients with MDD [35]. Most studies so far have been cross-sectional, and patients frequently received active pharmacological treatment prior to study inclusion (see review [35] for overview), which may have affected the gut microbiota as antidepressant medicine has been suggested to contain antibacterial properties [36, 37]. Furthermore, the gut microbiota has a key role in the development of the immune system [38] and in previous studies of the gut microbiota of patients with depression, inflammatory markers have been assessed, but not in treatment-naïve patients initiating treatment [39, 40]. Since antidepressants such as SSRIs have also been found to contain anti-inflammatory properties [41], there might be a gut microbiota– inflammation– antidepressant treatment triad that provide further insight into treatment options for patients with MDD. It is therefore important to assess the gut microbiota in treatment-naive patients to evaluate for specific gut microbiota and inflammatory profiles. Additionally, it is necessary to evaluate if subsequent changes in gut microbiota during antidepressant treatment are associated with MDD and changes in symptoms over time, or rather is an epiphenomenon linked to antidepressant treatment. The aim of this study was to conduct a characterization of the gut microbiota in untreated patients recently diagnosed with MDD before treatment initiation, including both antidepressant treatment and/or psychotherapy, and subsequently after four and twelve weeks of treatment, in comparison to a non-depressed group (nonMDD). Indices of α- and β-diversity, as well as significantly different relative abundance of phylotypes between patients with MDD and nonMDD controls, were the primary outcomes of the study. Secondary outcomes included changes in depressive symptoms, the inflammatory profile, as well as gastrointestinal symptoms and dietary habits.

## Methods

### Study design

This prospective cohort study investigated antidepressant-naive patients recently diagnosed with MDD compared to healthy individuals. The participants were assessed during a twelve week period with three sampling points: at baseline, and then four and twelve weeks later. The twelve week follow up was chosen to ensure that patients had received antidepressant treatment in a substantial enough time point to elicit a treatment response, and potentially a full remission, which has been observed in several studies to occur at around 2–4 weeks and at 12 weeks, respectively [42]. At each follow-up, fecal samples were collected, the severity of depressive symptoms measured with the major depressive inventory (MDI), and a questionnaire concerning gastrointestinal symptoms and diet was completed. Patients initiated antidepressant treatment and/or psychotherapy immediately after the baseline data was collected.

### Study population

Young adults aged 18 to 24 years were recruited from the Department of Psychiatry at Aalborg University Hospital, Aalborg and from the Psychiatric Department, Horsens Regional Hospital, Horsens, Denmark between December 22nd 2017 – March 13th 2020. Patients were screened and diagnosed according to the 10th revision of the International Classification of Diseases (ICD-10) [43] criteria for depressive episode (hereafter referred to as the MDD group) by a psychiatrist. Exclusion criteria were as follows: previous or current medical antidepressant treatment; psychiatric disorders other than MDD; neurological disorders; gastrointestinal disorders; endocrine, nutritional or metabolic diseases; infectious or parasitic diseases within a month prior to inclusion; use of antibiotics, probiotics, prebiotics or synbiotics within three months prior to inclusion; pregnancy within a year prior to inclusion; substance or alcohol abuse; and specific dietary habits, such as vegetarian or paleolithic diets (see the complete list of exclusion criteria in Supplementary Material 1).

Non-depressed individuals (hereafter referred to as the nonMDD group) aged 18 to 30 years were recruited through social media, and on the intranet webpage of the North Denmark Regional Hospital. Exclusion criteria were identical to those for patients with MDD, in addition to no current or previous history of psychiatric disorders or antidepressant use. Verification of the medical history of both MDD and nonMDD participants was performed retrospectively through data retrieved from the Danish electronic patient journal system at a minimum of one year after the last patient with MDD was recruited for the study.

### Questionnaires and data acquisition

#### Major depressive inventory

At each sampling point, all participants were instructed to use a self-screening 10-item MDI questionnaire to evaluate severity of their depressive symptoms [44]. Based on total scores, participants were grouped into four categories: no depression (0–20), mild depression (21–25), moderate depression (26–30) and severe depression (31–50).

#### Bristol Stool Scale

Participants were asked to rate their stool sample consistency using the Bristol Stool Scale (BSS), which was used as a proxy for bowel transit time [45]. The instrument consists of seven points, with a 1 being stool with long transit time representing constipation and a 7 being very short transit time representing diarrhea. Participants were instructed to collect a fecal sample only if the BSS score of the sample was between 3 and 5, as severely decreased or increased transit time would affect the gut microbiota composition [46].

#### Demographic and clinical data

At inclusion, participants gave information on their age, height, weight, and smoking habits. Participants were asked to fill out a baseline questionnaire with focus on appetite, dietary habits, bowel movements, and gastrointestinal symptoms. Questions included the number of daily meals, estimation of appetite and self-evaluation of their caloric intake in accordance with Danish guidelines of nutritious and healthy eating. Additionally, they were asked about toilet habits. In the questionnaire participants received during the four- and twelve-weeks follow-ups, the questions focused on changes experienced in the parameters compared to the baseline measurements.

### Microbiota characterization by 16 S rRNA gene sequencing

Participants were instructed by study personnel, both orally and with a written manual, on how to collect, store and transport stool samples. They received a collection kit containing a cooling bag, cooling blocks, gloves, sterile tubes, and an Easy Sampler collector (GP Medical Devices ApS) to ensure easy and hygienic collection without contamination from surroundings. Fecal samples were collected in the homes of the participants and immediately stored in a domestic freezer (-20 °C) for a maximum of 72 h until delivery in a cooling bag to the laboratory. Following reception at the laboratory, fecal samples were stored at -80 °C. Total DNA was isolated from 250 ± 25 mg fecal samples, using the QIAamp Powerfecal DNA kit (QIAGEN) with automation on a QIAcube® (QIAGEN) as previously described [47]. 16 S rRNA gene sequencing was performed by DNAsense ApS Denmark as previously described [48]. In brief, sequencing libraries were constructed using a standardized primer set (515 F(Parada) and 806R(Apprill) [49, 50]) targeting the 16 S rRNA V4 region. This was followed by a PCR where unique barcoded primers were added to all sequencing libraries. The resulting DNA was sequenced (2 × 300 bp) on a MiSeq platform using MiSeq Reagent kit V3 (Illumina) with an additional 10% PhiX control library (Illumina) to estimate error rate during sequencing.

### Measurement of blood plasma biomarkers

Within 24 h of collection of the fecal sample (+/-), Blood was drawn from a peripheral vein at all three time points (at baseline, and at 4 and 12 weeks follow up) within 24 h (+/-) of collection of the fecal sample.,Plasma was isolated by centrifugation and stored at − 80 °C until further analysis. Lipopolysaccharide-binding protein (LBP) was used as a proxy for the concentration of LPS [51]. LBP was measured in peripheral blood plasma samples in duplicates with the RayBio® Human LBP ELISA Kit (RayBiotech, USA) according to manufacturer’s instructions. All incubation steps were performed at room temperature using an orbital shaker (Thermo Fisher Scientific) set to 150 rpm. Signal intensity was measured at 450 nm for LBP on a Fluostar Omega Plate Reader (BMG Labtech, Germany). Concentrations were calculated using the four-parameter logistic regression method, as per manufacturer’s recommendation.

Immune markers were analyzed in blood plasma, using the electrochemiluminescent immunoassays Mesoscale Diagnostics technology to evaluate the degree of systemic inflammation in the participants. A combination of six panels were used, namely the V-PLEX Angiogenesis Panel 1 Human (basic FGF, PIGF, Tie-2, VEGF-A, VEGF-C, VEGF-D, VEGFR-1/Fit-1), V-PLEX Chemokine Panel 1 Human (Eotaxin, Eotaxin-3, IL-8, IP-10, MCP-1, MCP-4, MDC, MIP-1α, MIP-1β, TARC), V-PLEX Cytokine Panel 1 Human (IFN-γ, IL-1β, IL-2, IL-4, IL-6, IL-8, IL-10, IL12p70, IL-13, TNF-α), V-PLEX Cytokine Panel 2 Human (IL-1RA, IL-3, IL-9, IL-17 A/F, IL-17B, IL-17 C, IL-17D, TSLP), V-PLEX Proinflammatory Panel 1 Human (IFN-γ, IL-1 β, IL-2, IL-4, IL-6, IL-8, IL-10, IL-12p70, IL-13, TNF-α), and V-PLEX Vascular Injury Panel 2 Human (CRP, ICAM-1, SAA, VCM-1). Analyses were performed at the Department of Clinical Immunology, Copenhagen University Hospital, Denmark according to manufacturer’s protocol. Positions on the plates were randomized and samples analyzed in triplicates. Results were log2 transformed and displayed as fluorescent intensity. To adjust for variations across plates, signal intensities were median-normalized across the individual plates. For both ELISA and Mesoscale measures, assay diluent was used as negative control. Signals below that of blank controls were included into the analyses.

### Bioinformatics

PhiX sequences were removed and read pairs were demultiplexed by the USEARCH v.11 pipeline [52]. The resulting demultiplexed sequences were imported into the QIIME2 2020.8 bioinformatics platform [53]. To build amplicon sequence variants (ASVs), primers were filtered from forward reads, followed by truncation to 250 base pairs and denoising with DADA2 using standard parameters. All samples had > 10.000 reads, with a large gap to the majority of negative controls (Supplementary Fig. 1) and were thus included in the final analysis. All ASVs were aligned with MAFFT [54] using q2-alignment, and a phylogenic tree was constructed hereof using fasttree2 [55] implemented in q2-phylogeny. Taxonomy was assigned using the Naïve Bayesian classifier, implemented in q2-feature-classifier, trained against the SILVA 138 SSU reference database [56]. R version 4.0.3 was used for subsequent analyses through the Rstudio IDE (http:///www.rstudio.com). The generated amplicon data was investigated using the packages phyloseq v1.32.0 and ampvis2 v2.6.6. α-diversity metrics were analyzed using ASV richness, Faith’s phylogenic diversity, and Shannon diversity index. β-diversity indices included principal coordinate analysis (PCoA) of Bray-Curtis dissimilarity, as well as weighted and unweighted UniFrac [57]. Bacterial β-diversity was analyzed using permutational multivariate analysis of variance (PERMANOVA) with 999 permutations, as implemented in ADONIS and tested for variability using Betadisper. The Analysis of Compositions of Microbiomes with bias correction (ANCOM-BC) [58] was used to determine the most differentially abundant taxa between the different diagnostic groups, or time points.

### Statistical analyses

For continuous data such as age, BMI, LBP and cytokine concentrations, distribution and variance were determined using Shapiro-Wilks test and Bartlett’s test, respectively. Baseline α-diversity differences as well as differences in LBP and cytokines between MDD and nonMDD were tested using either Student’s t-test or Mann-Whitney U test, depending on normality. To analyze changes in α-diversity over time, we used the repeated measure ANOVA. For paired data, such as the MDI score and longitudinal measurements of LBP and cytokines, a mixed effect model was used to account for missing data. For correlation analysis between inflammatory markers, LBP, MDI and β-diversity measures, Spearman’s rank correlation coefficient was used, and the monotony of the correlation was visualized using scatterplots. For univariate statistics, the null hypothesis was rejected if *p* < 0.05, whereas for multivariate statistics, a Benjamini-Hochberg adjusted *p*-value (q) < 0.05 was used. Sample size was based on previous studies showing differences in microbiota compositions between cases and controls with between 10 and 17 cases included [59–61], indicating that our sample size could be expected to be sufficient for this purpose.

### Ethics

The study was conducted in accordance with the Declaration of Helsinki and approved by the North Denmark Regional Ethical Committee (reference: N-20,170,056). The categories of the personal data collected in the project were registered in the processing activities of research in the North Region of Denmark in compliance with EU GDPR article 30. Written and oral informed consent was obtained from all participants.

## Results

**Fig. 1:**
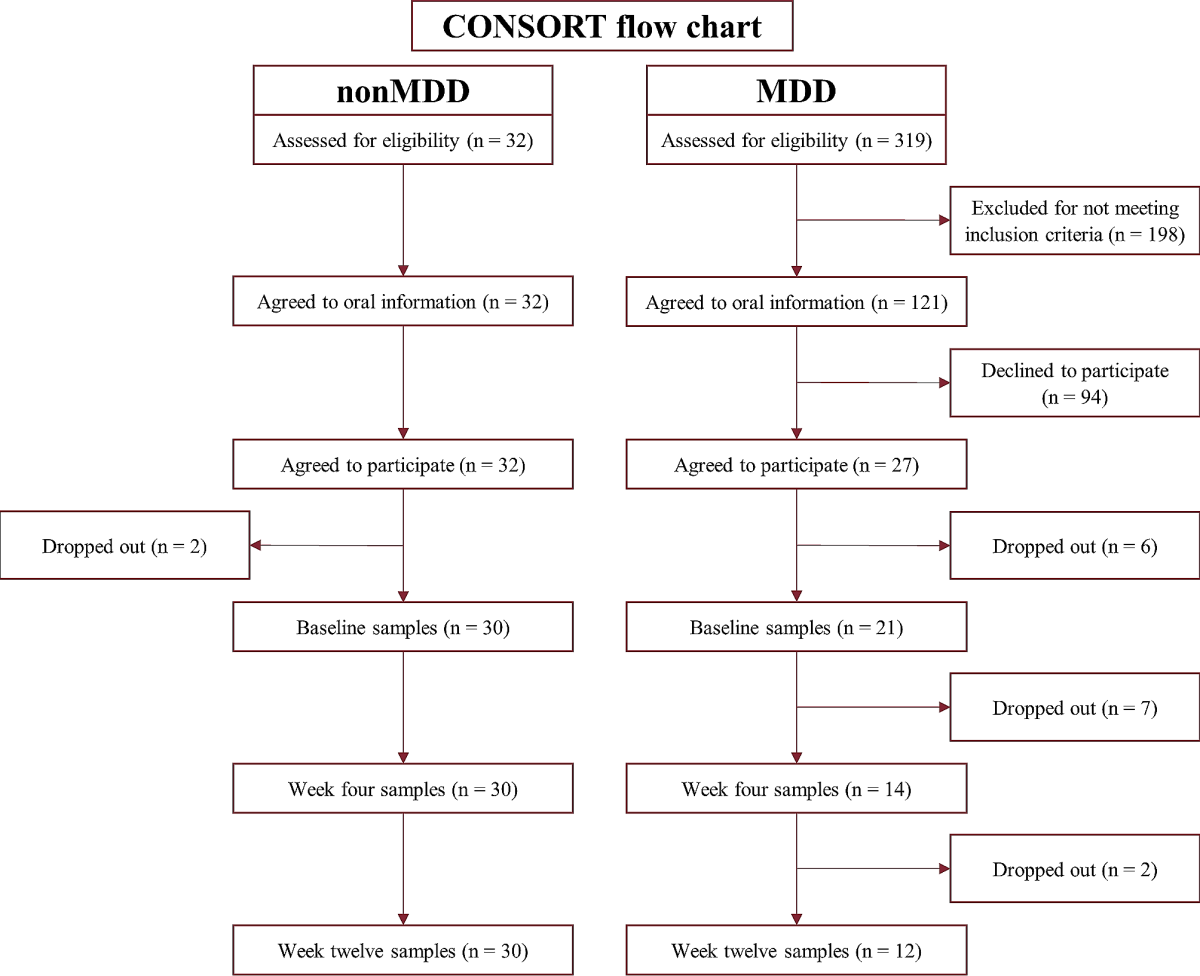
CONSORT flow diagram displaying the process of recruitment and adherence to the study

In total, 27 individuals in the MDD group and 32 individuals in the nonMDD group agreed to participate in the study (see Fig. 1). Of these, 21 (78%) in the MDD group and 30 (94%) in the nonMDD group delivered the baseline samples, and 13 (48%) in the MDD group and 30 (94%) in nonMDD group completed the study by attending both follow-ups.

Demographic and clinical data are presented in Table 1. There was no significant difference between the MDD and nonMDD groups in BMI, sex or smoking. However, there was a significant difference in age (MDD = 20.9 ± 4.2 and nonMDD = 23.7 ± 10.7, *p* < 0.001). Most participants in the MDD group commenced antidepressant treatment, but two individuals received psychotherapy only during the study. Antidepressant treatment consisted primarily of serotonin reuptake inhibitors, albeit in three individuals the atypical antipsychotic quetiapine was added before the twelfth week follow-up. Compared to the nonMDD group, the MDD participants displayed apparent depressive symptoms with a significantly higher MDI score (MDD = 40.3 ± 6.9 versus nonMDD = 4.9 ± 4.2, *p* < 0.001) at baseline. Overall, there was a trend towards lower MDI score in the MDD group at the four weeks follow-up (35.5 ± 8.2, *p* = 0.13) and a significant difference in MDI score at the twelve weeks follow-up (29.4 ± 12.1, *p* = 0.02) compared to the MDI score at baseline. After twelve weeks, two in the MDD group had a MDI score lower than 20, indicating remission, and two reported a minimum of 50% reduction in MDI score, indicating response to treatment. Furthermore, five participants went from moderate to mild depression based on their MDI score, indicating a partial response to treatment.

The MDD group reported gastrointestinal symptoms, such as constipation, bloating and stomach pain, at a higher frequency than the nonMDD group throughout the study (baseline measurements: MDD = 48% and nonMDD = 10%, *p* < 0.001). However, there was no significant difference between the two groups in BSS score (*p* = 0.85). Participants were instructed to evaluate their general calorie intake in comparison to general Danish guidelines, as well as subjective feeling of appetite at baseline, and to evaluate whether they experienced any changes in these two parameters during the twelve weeks they participated. One participant in the MDD group (7%) and three participants in the nonMDD group (10%) reported slight changes in appetite or caloric intake during the twelve week study (data not shown).

**Table 1:** Demographic and clinical data at inclusion and during the study period

Demographic and clinical data for participants during the study	MDD				nonMDD				MDD vs. nonMDD
	Baseline	4 weeks	12 weeks	*p*-value	Baseline	4 weeks	12 weeks	*p*-value	*p*-value
Age (years)	20.9 ± 4.2				23.7 ± 10.7				0,001
Gender (% female)	76% (16/21)				83% (25/30)				0,66
BMI (kg/m2)	24.4 ± 5.1				23.0 ± 2.8				0,168
Smoking (yes)	20% (4/21)				20% (6/30)				0,914
MDI	40.3 ± 6.9	35.5 ± 8.2	29.4 ± 12.1	*0.13 / § 0.02	4.9 ± 4.2	3.7 ± 2.7	5.0 ± 4.4	*0.03 / § 0.45	< 0.0001
Active antidepressant medical treatment (yes)	0% (0/21)	85.7% (12/14)	83.3% (10/12)		0% (0/30)	0% (0/30)	0% (0/30)		
Gastrointestinal symptoms (yes)	50% (10/21)	64. % (9/14)	58.3% (7/12)		10% (3/30)	6.7% (2/30)	10% (3/30)		
Bristol Stool Scale Score	3.5 ± 0.6	3,3 ± 1,0	3.4 ± 1.2	*0.18 / § 0.53	3.5 ± 0.6	3.7 ± 0.6	3.7 ± 0.8	*0.33 / § 0.41	0,85

Values are presented as mean (± SD) or as percentages (n/total). Gastrointestinal symptoms covered stomach pain, constipation, nausea, and diarrhea. * Comparison between baseline and four weeks samples. § comparison between baseline and twelve weeks samples. BMI: Body mass index. MDI: Major depressive inventory

### Quality assessment of 16 S rRNA gene sequencing

Sequencing of the 16 S rRNA gene gave rise to a total of 6,440,258 reads, generating 2,083 ASVs with a median of 47,301 reads per sample. The ASVs could be assigned taxonomically to bacterial phylum (99.4%), family (95.3%) and genus (87.4%). In the MDD group, a total of 1,247 unique phylotypes were observed, compared to 1,657 in the nonMDD group. The distribution of the mean reads and rarefaction curves can be viewed in Supplementary Fig. 1.

### α- and β-diversity indices between MDD and nonMDD at baseline and after initiation of antidepressant treatment

**Fig. 2:**
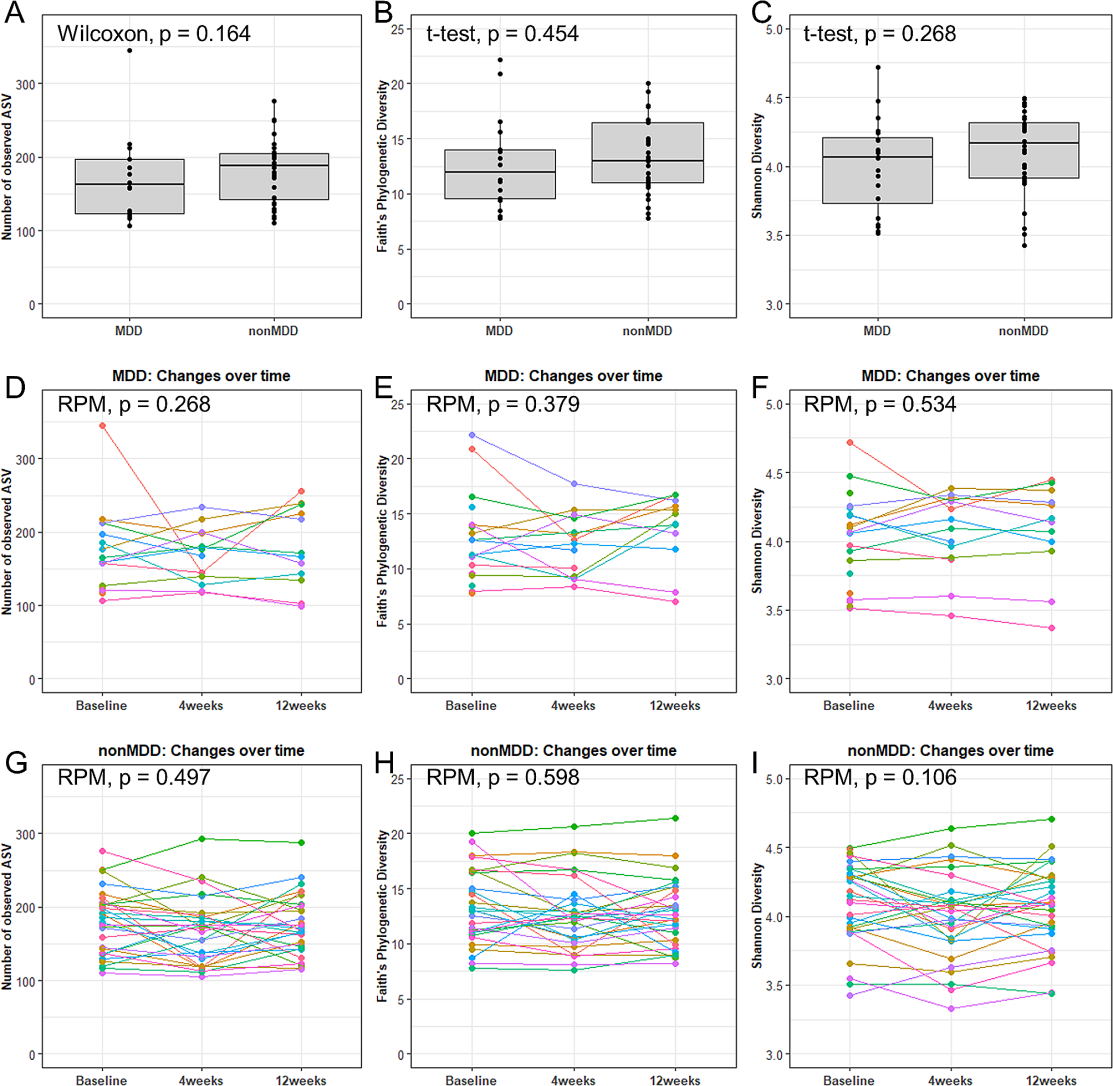
α-diversity in untreated patients with MDD and compared to healthy individuals (nonMDD). Number of observed amplicon sequence variants (ASVs) (**A**, **D**, **G**), Faith’s phylogenetic diversity (**B**, **E**, **H**) and Shannon diversity index (**C**, **F**, **I**) as compared between MDD and nonMDD (**A**, **B**, **C**), in-between samples of MDD collected over time (**D**, **E**, **F**) and in-between samples of nonMDD collected over time (**G**, **H**, **I**)

Differences in α-diversity of gut microbiota between MDD and nonMDD groups at baseline and over time was analyzed using the number of observed ASVs, Faith’s phylogenetic diversity and Shannon diversity index. There was no difference in α-diversity between the MDD and nonMDD group at baseline (Fig. 2A-C). Likewise, no significant change in α-diversity was observed for either group at four or twelve weeks follow-up (Fig. 2D-I). When comparing MDD to nonMDD at four and twelve week follow up, no significant change was observed (Supplementary Fig. 2 for α-diversity and Supplementary Fig. 3 for β-diversity).

**Fig. 3:**
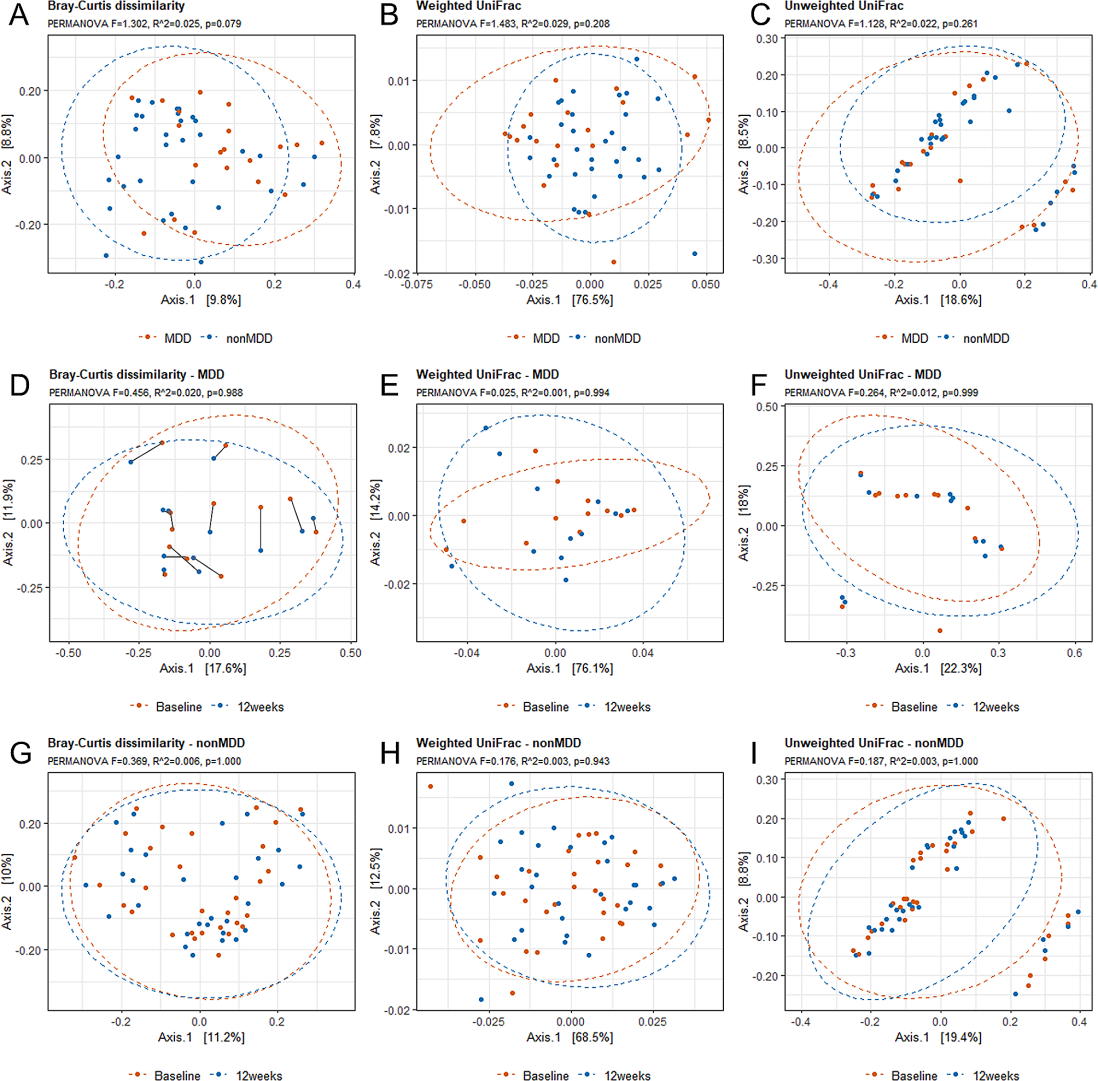
Gut microbiota β-diversity. Bray-Curtis dissimilarity (**A**, **D**, **G**), weighted UniFrac (**B**, **E**, **H**) and unweighted UniFrac (**C**, **F**, **I**) as compared between MDD and nonMDD (**A**, **B**, **C**), within the MDD group with samples collected at baseline and at twelve weeks (**D**, **E**, **F**) and within the nonMDD group with samples collected at baseline and at twelve weeks (**G**, **H**, **I**)

We then explored if it was possible to separate MDD from nonMDD based on β-diversity measures using Bray-Curtis dissimilarity distance, as well as weighted and unweighted UniFrac. Neither measure showed group-specific clustering of gut microbiota (Fig. 3A-C). Furthermore, we did not observe any changes in β-diversity at four and twelve weeks follow-up in neither the MDD group (Fig. 3D-F) nor the nonMDD group (Fig. 3G-I). Overall bacterial composition between sampling points, at genus and phylum level, is furthermore depicted in heatmaps and bar plots (Supplementary Figs. 4 and 5).

### Bacterial taxa between MDD and nonMDD at baseline and after initiation of antidepressant treatment

**Fig. 4:**
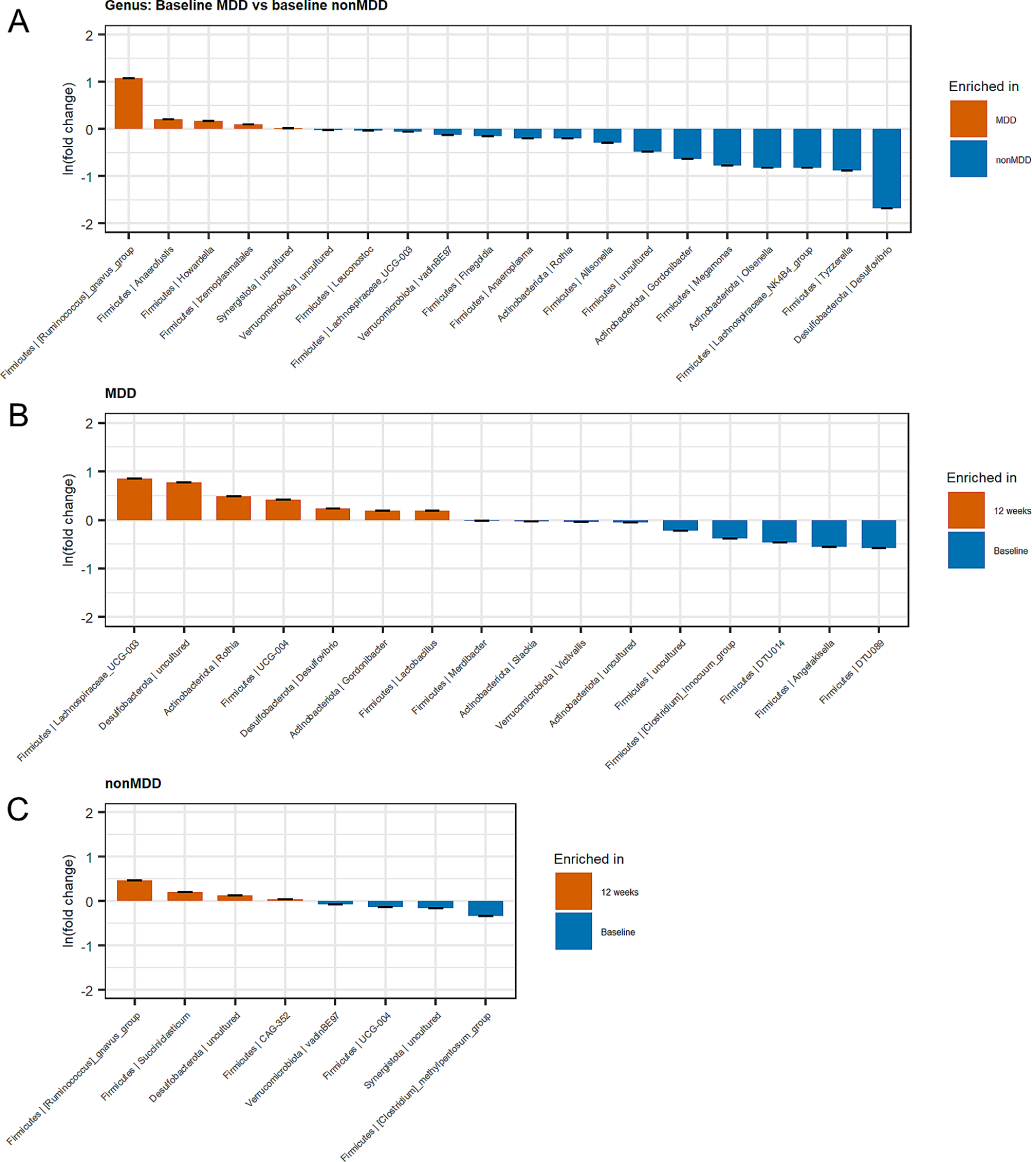
ANCOM-BC analysis of individual bacterial taxa changes. Bar plots display the log fold change in relative bacterial taxa compared between the MDD group versus the nonMDD group at baseline (**A**), between the MDD group at baseline versus 12 weeks follow-up (**B**), and between the nonMDD group at baseline versus 12 weeks follow-up (**C**)

Several orders, families and genera of bacteria were observed to be significantly different in relative abundance between the MDD and nonMDD participants at baseline. In total, 20 different taxa were found to be significantly different (Fig. 4A). For example, the genera *Ruminococcus gnavus* group, *Anaerofustis, Howardella*, and *Izemoplasmatales* were increased in relative abundance in the MDD group compared to the nonMDD group. In contrast, the genera *Desulfovibrio, Tyzzerella, Olsenella, Megamonas, Gordonibacter, Allisonella, Rothia, Anaeroplasma* and *Finegoldia* were observed to be decreased in relative abundance in the MDD group compared to the nonMDD group. After twelve weeks, several phylotypes were observed to change significantly in relative abundance in the MDD group compared to baseline. There were among others an increase in relative abundance in the genera *Rothia, Desulfovibrio, Gordonibacter* and *Lactobacillus*, and a decrease in the genera *Angelaksiella, Clostridium* inoculum group, *Victivallis, Slackia* and *Merdibacter* (Fig. 4B). The nonMDD group also displayed changes at twelve weeks follow-up, although to a lesser extent, with an increase in relative abundance of the genera *Ruminococcus* gnavus group and *Succiniclasticum*, and a decrease in relative abundance of the genus *Clostridium* pentosum group (Fig. 4C).

### Inflammatory profiles between groups and over time

**Fig. 5:**
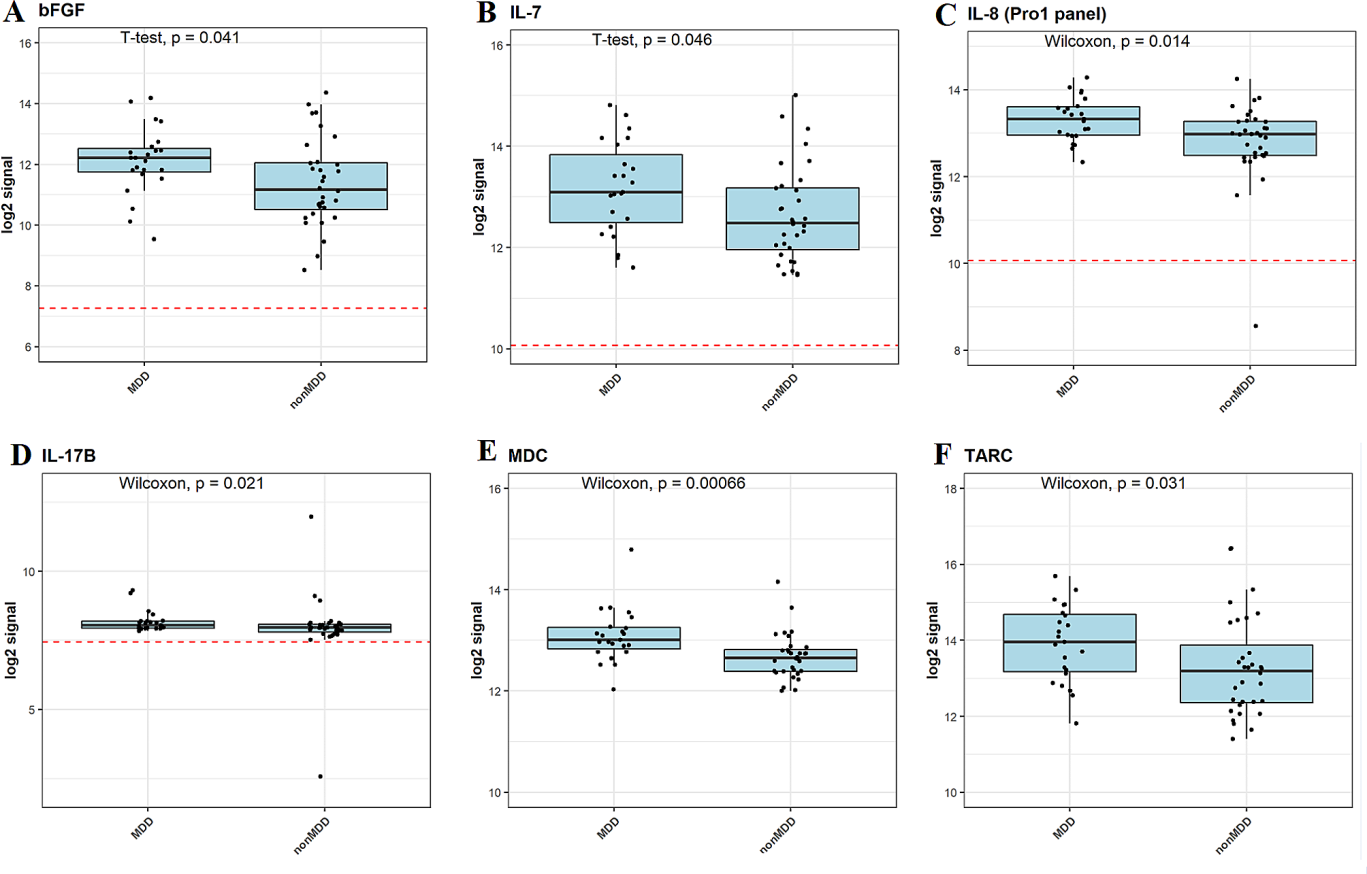
Plasma inflammatory markers significantly different between the MDD and nonMDD groups. The red dashed line represents the log2 signal intensity of the buffer control. Measurements with a signal below this cutoff line were included in the statistical analyses

**Fig. 6:**
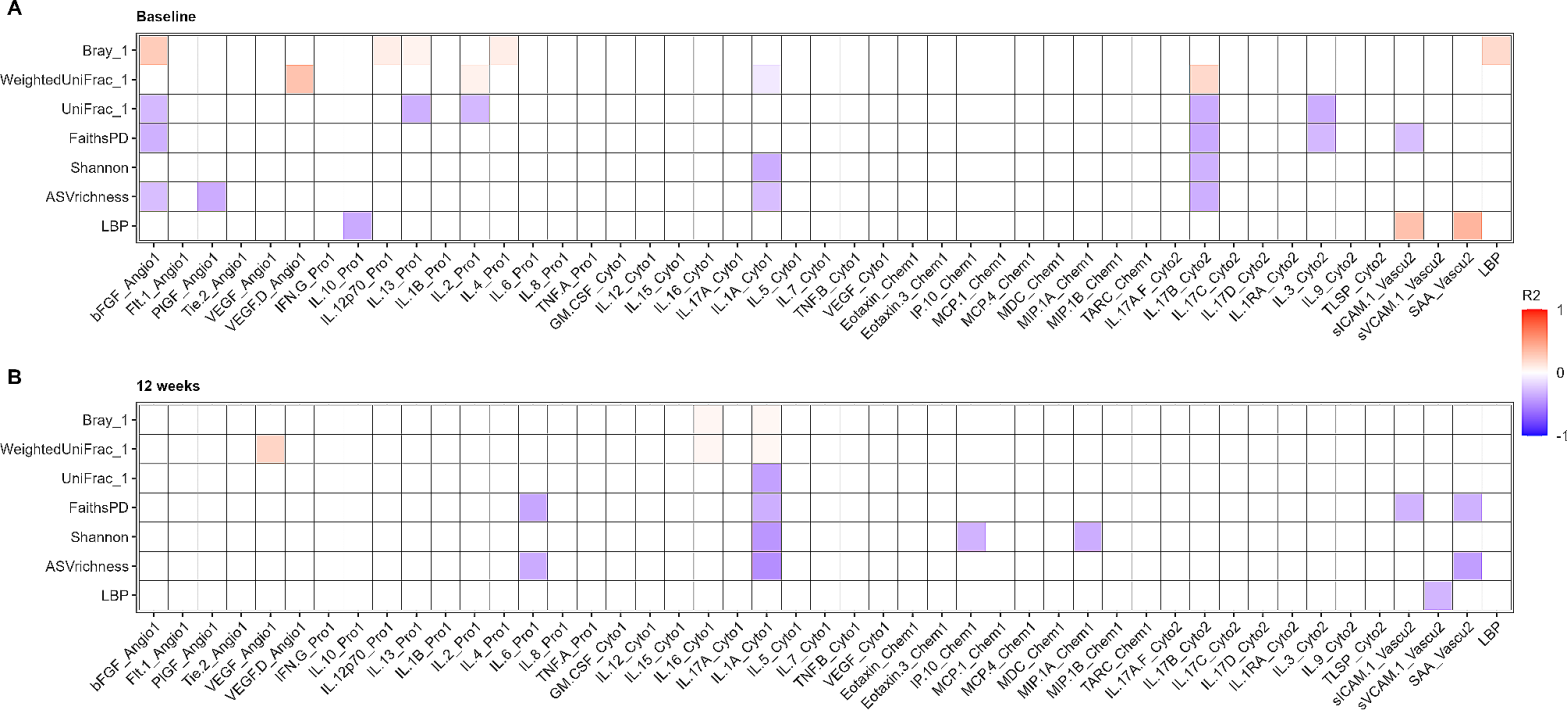
Correlation matrix of microbiota markers and LBP compared to plasma immune markers. Correlations were tested using Spearman’s correlation. Red boxes indicate positive correlations ($$ {R}^{2}$$>0), while blue boxes indicate negative correlations ($$ {R}^{2}$$<0). Blank boxes indicate that correlations did not reach significance

At baseline, the MDD group was found to have significantly increased levels of several cytokines in comparison to the nonMDD group (Fig. 5A-F). In plasma, basic fibroblast growth factor (bFGF, *p* = 0.041), interleukin-7 (IL-7) (*p* = 0.046), IL-8 (*p* = 0.014), IL-17b (*p* = 0.021), macrophage-derived chemokine (MDC, *p* < 0.001), and thymus and activation regulated chemokine (TARC, *p* = 0.031) were elevated in the MDD group compared to the nonMDD group. This would suggest a systemic pro-inflammatory profile. No significant difference was found between the MDD and nonMDD groups in the remaining inflammatory markers, or in the LBP measurements (data not shown). None of the inflammatory markers significantly different between the MDD and nonMDD groups were found to decrease during antidepressant treatment in the MDD group (Supplementary Fig. 6). Correlation analyses showed that some of the inflammatory markers, found to be significantly different between the MDD and nonMDD groups at baseline, correlated with overall β-diversity in both groups (Fig. 6A). We observed that bFGF was positively associated with Bray-Curtis dissimilarity (*p* = 0.024), but negatively associated with unweighted UniFrac, ASV richness, and Faith’s phylogenic diversity (*p* = 0.033, *p* = 0.046 and *p* = 0.016, respectively). Additionally, IL-17b was negatively associated with both weighted and unweighted UniFrac, as well as ASV richness, Faith’s phylogenic diversity, and Shannon diversity index (*p* = 0.012, *p* = 0.011, *p* = 0.012, *p* = 0.008 and *p* = 0.017, respectively). LBP concentrations correlated positively with Bray-Curtis dissimilarity (*p* = 0.027), soluble intercellular adhesion molecule-1 (sICAM-1, *p* = 0.043), and serum amyloid A (SAA, *p* = 0.005) and negatively with IL-10 (*p* = 0.008). Furthermore, weighted UniFrac correlated positively with VEGF-D, and negatively with IL-1a. Only the positive correlation between weighted UniFrac and VEGF-D, as well as the negative correlations between IL-1 A and ASV diversity and Shannon diversity index, were maintained after twelve weeks (*Figur 6B).*

### Interactions between MDD symptoms, the gut microbiota and inflammation

**Fig. 7:**
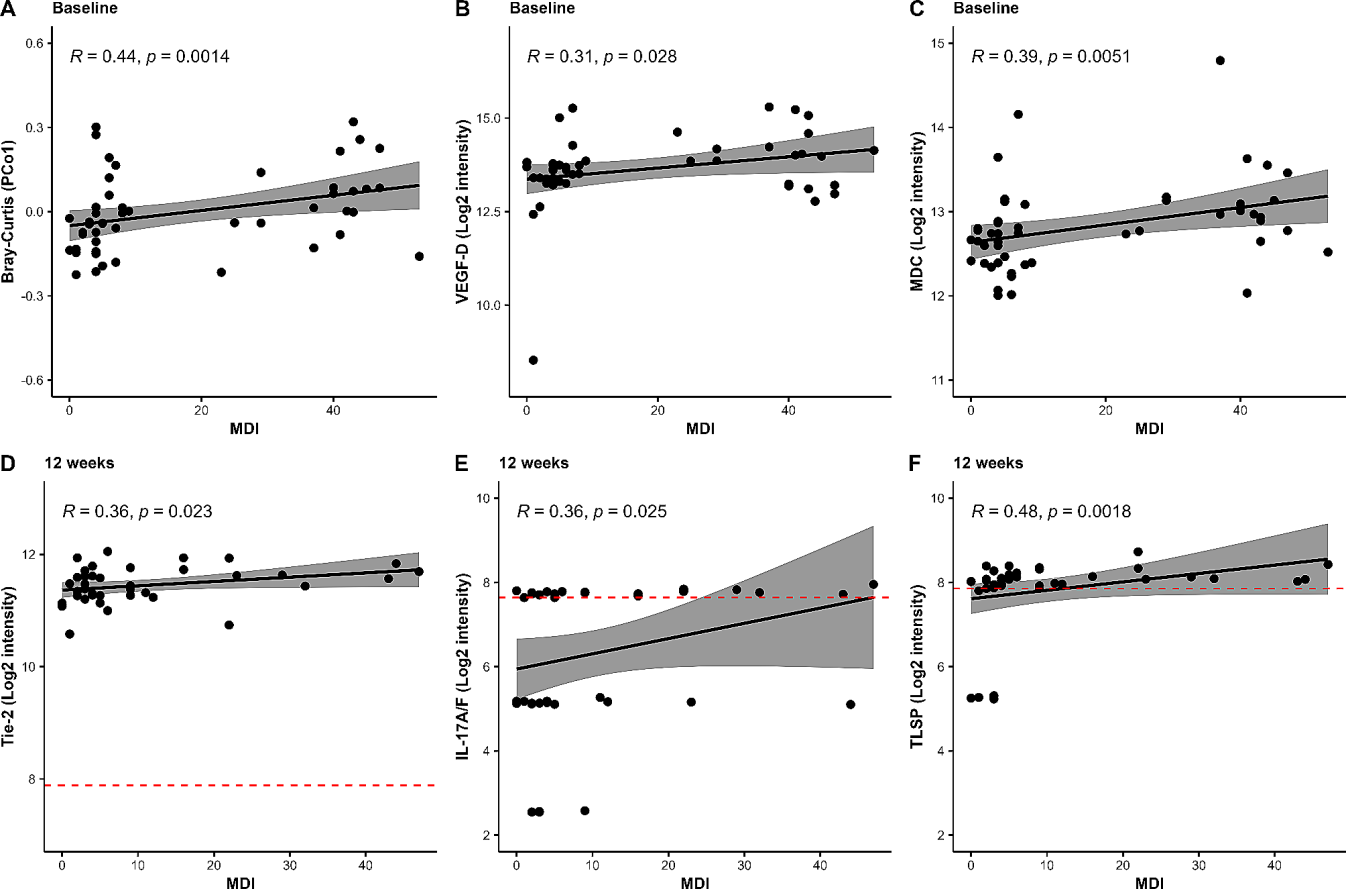
Correlation analysis between MDI score, gut microbiota measures and inflammatory biomarkers. Correlation was tested using Spearman correlation at baseline and after 12 weeks. Only those correlations that reached significance are depicted. The red dashes represent the log2 signal intensity of the buffer control. Measurements with a signal below this cutoff line were included in the statistical analyses

After assessing the gut microbiota composition, inflammatory markers and MDI score, we wanted to explore if there was a correlation between these different parameters, as well as analyze if these changed during the antidepressant treatment. A correlation analysis was performed, which revealed a positive correlation between MDI and Bray-Curtis dissimilarity (*p* = 0.001), VEGF-D (*p* = 0.028) and MDC (*p* = 0.005) (Fig. 7A-C). This, in combination with the other results, suggests that there is a correlation between depressive symptoms, intestinal bacterial taxa and inflammation markers before initiation of pharmacological/cognitive antidepressant treatment, although it was not maintained for the same inflammatory markers following treatment (Fig. 7D-F).

## Discussion

In this study, we characterized the gut microbiota in antidepressant-naive patients with MDD and compared them with a non-depressed group. The MDD group then started antidepressant treatment and/or psychotherapy and samples were collected again at four and twelve weeks follow-up. At baseline, we found no difference between the MDD and the nonMDD group in α- or β-diversity, but individual bacterial taxa were significantly different between the two groups. An increased relative abundance of the genera *Ruminococcus gnavus* group was observed, while the genera *Desulfovibrio, Tyzzerella, Olsenella, Megamonas, Gordonibacter, Allisonella* and *Rothia* were decreased when comparing the MDD group to the nonMDD group. Furthermore, it was found that the MDD group had a pro-inflammatory profile consisting of increased bFGF, IL-7, IL-8, IL-17b, MDC and TARC, and at the same time, several inflammatory biomarkers were correlated with bacterial parameters. This included fFGF that correlated positively with Bray-Curtis dissimilarity, and negatively with unweighted UniFrac, ASV richness, Faith’s phylogenic diversity. Furthermore, MDI was observed to correlate positively with Bray-Curtis, VEGF-D and MDC at baseline, and with Tie-2, IL-17 A/F and TLSP following twelve weeks of treatment.

Previous studies of gut microbiota in patients with MDD have reported heterogenous results on which bacterial taxa that were observed to be significantly altered in relative abundance between MDD and nonMDD groups [62]. In our population of patients with MDD, overrepresentation of *Ruminococcus* was observed, which has also been reported previously [63], However, other studies have observed underrepresentation compared to healthy controls [59, 64, 65]. The same was observed for *Howardella* which was increased in this study, but decreased in relative abundance in another study [66]. Likewise discrepancies were observed for bacteria reduced in our MDD group, as decreased *Desulfovibrio, Megamonas and Gordonibacter* was in accordance with some studies ([13, 63, 64] and [65] (males) for each respective taxa), but in contrast with others ([59, 66, 67] and [66] (females) for each respective taxa). *Olsenella* and *Rothia* were observed depleted in our MDD group, but increased in other studies ([34, 67, 68] and [34] for each respective taxa). The only consistency was *Tyzzerella*, which was observed to be depleted in the MDD group in our study, as well as one other study [64]. The remaining genera *Anaerofustis, Izemoplasmatales, Allisonella, Anaeroplasma* and *Finegoldia* have not previously been reported altered in patients with MDD to our knowledge and may therefore represent population-specific taxa unique to our cohort. Some of the bacteria observed to be elevated or depleted in the MDD group here and in previous publications may contain depressogenic or antidepressant properties, respectively. Overrepresentation of the *Ruminococcus* genus has previously been found in a study by Lukíc et al. to induce down-regulation of genes involved in neuronal plasticity in mice [69], a recognized neurobiological feature of MDD [70]. Additionally, the *Ruminococcus gnavus* group can metabolize tryptamine from tryptophan [71], limiting the production of serotonin from tryptophan, and thereby the bioavailability of this neurotransmitter that is important in the treatment of MDD. Acute tryptophan depletion has been linked to exacerbation of depressive symptoms in patients in remission [72]. The reduced *Desulfovibrio* can also be linked to neurotransmitter production, as a study in the depression rat model exposed to chronic mild stress, found a positive association between neurotransmitters in the hippocampus, anti-depressive behaviors and the relative abundance of *Desulfovibrio* [73]. Increased relative abundance of *Ruminoccocus* and decreased relative abundance of *Desulfovibrio* may therefore combined lead to low neurotransmitter production.

There is a lack of consensus regarding diversity indices and specific bacteria observed at baseline in patients with MDD in this study and previous publications, which has been a generalized problem in the field of gut microbiota association with MDD [35]. The studies conducted so far span different geographical regions, whereby it is possible that the lack of consensus between studies arises from dietary preferences and their influence on the gut microbiota composition [74–76]. Additionally, previous studies have included a wide age group (often spanning between 18 and 65 years of age), by which the risk of comorbid disorders and diseases increase. Noticeably, few studies excluded patients or controls with other psychiatric disorders than MDD or comorbid somatic disorders [35]. Several diseases such as inflammatory bowel disorders [77] and type 2 diabetes [78] have been found to contain significantly different gut microbiota compared to healthy individuals. This may have resulted in alterations in the gut microbiota composition by bacterial species associated specifically with the comorbid disorder, masking identification of bacterial taxa or bacterial diversity unique to MDD. In the assessment of changes in bacterial diversity indices, it was not possible to separate the overall gut microbiota of patients with MDD before antidepressant treatment and after twelve weeks of antidepressant treatment and/or psychotherapy. Due to the low sample size, the microbial signature between responders and non-responders was not evaluated. Nevertheless, there were several bacterial taxa which changed in relative abundance during the twelve weeks study, with an increase in *Desulfovibrio, Rothia, Gordonibacter* and *Lactobacillus* and a decrease in the family *Ruminococcaceae* and the genera *Angelaksiella, Clostridium* innocuum group, *Victivallis*, and *Slackia* after twelve weeks of treatment.

To our knowledge, this is the first study to characterize the immune profile in combination with the gut microbiota in patients with MDD. Large-scale meta-analyses have observed a different immune profile than in this present study [79]. However, studies agree with some of our observations, such as elevated IL-7 and bFGF [80], and elevated IL-17b in patients with MDD compared to healthy controls [81]. On the other hand, Il-7 and IL-8 have previously been reported decreased in treatment-naive patients with MDD compared to healthy controls ([82] and [83], respectively), where we observed the opposite in our study. Increased pro-inflammatory cytokines are associated with elevated macrophage activity promoting depressive symptoms [84], and has also been found elevated in previous studies of assessments of gut dysbiosis in patients with MDD [41]. IL-7 is both an active chemokine for macrophage recruitment, as well as in macrophage differentiation [85], and macrophages have been found to contain a pro-inflammatory profile in depression [86]. As MDC and IL-8 are excreted by macrophages [87, 88], and TARC promotes an M2 profile [89], our findings could indicate a dysregulation of macrophage profile and activity, which has been proposed before in MDD pathogenesis [90]. The gut microbiota has been found to regulate immune homeostasis [39], such as mediating an M2 profile [91], and combined with the elevated immune factors in our study of patients with MDD, this suggest a link between the elevated inflammatory markers, gut dysbiosis and depressive symptoms. This is furthermore supported by our correlation analysis, where we found that LBP was correlated to many of the inflammatory factors, such as diversity using weighted UniFrac, as well as SAA, sICAM and IL-10. As the function of LBP is detection of LPS, the Gram-negative cell wall component [51], correlation between LBP and inflammatory factors suggest that these increases may be linked to an altered gut microbiota. This has been suggested in a previous study where elevated LBP in patients with MDD was associated with an abnormal monocyte profile [92]. In another study, increased production of SAA was associated with increased filamentous bacteria in the gut of mice in a study of induced Th17-mediated MDD [93]. As LBP was also negatively correlated with the anti-inflammatory IL-10, which has previously been found decreased in patients with MDD [94], this suggests that the gut microbiota confers a pro-inflammatory profile in patients with MDD.

We found that bFGF was positively associated with Bray-Curtis dissimilarity, but negatively associated with unweighted UniFrac, ASV richness, and Faith’s phylogenic diversity. While Bray-Curtis dissimilarity is based on presence or absence of bacteria, weighted for abundance, unweighted UniFrac is not weighted, but takes the internal phylogenic relationship into account [95]. Taken together, this may indicate that a high bFGF is associated with variation in several low-abundant ASVs, but not among the high abundant ASVs, indicating that unique species may be associated with depressive symptoms. A large meta-analysis of 27 studies on patients with MDD receiving probiotics found a significant reduction in depressive symptoms [96]. Furthermore, an altered gut microbiota has been implicated in inflammatory bowel diseases such as irritable bowel syndrome [97], ulcerative colitis and Crohn’s disease [98] and these diseases additionally harbor a higher risk of developing depression [99], strengthening the hypothesis of a depression-inflammation-gut microbiota triad [100], as well as supports the hypothesis of the gut-brain axis involvement in MDD [101]. This was further underlined by our analysis of MDI score at baseline, and its correlation to both microbial parameters as well as inflammatory biomarkers. Here, we found that MDI was positively correlated to Bray-Curtis dissimilarity, as well as VEGF-C and MDC. As MDI was correlated to Bray-Curtis dissimilarity, but neither weighted nor unweighted UniFrac, the variation appears to be among closely associated ASVs. The association between MDI and immune markers were not constant over time and after twelve weeks, MDI was positively correlated to Tie-2, IL-17 A/F and TLSP. The effect of antidepressant treatment on inflammatory markers has previously been assessed [102], but the results did not agree with our findings. However, since MDI was positively associated with IL-17 A, and this cytokine has previously been linked to treatment resistance [103], this may explain a connection between MDD and the gut microbiota-regulated IL-17 A production and function [104]. This also ties in with our previous theory of gut microbiota-associated dysregulated macrophage and T-cell function given the elevated inflammatory markers and their association with LBP. Additionally, MDI scores also correlated with MDC, which was elevated in MDD compared to nonMDD. MDC has previously been found to be increased in patients with MDD who responded to pharmacological treatment compared to before initiation of treatment [105]. This suggests that inflammatory parameters together with intestinal taxa might in combination be a potential biomarker for pharmacological antidepressant response.

Previous studies have examined antidepressant treatment in patients with MDD with characterization of the gut microbiota [106–109]. There was no overall consensus between these studies analyzing gut microbiota alterations caused by antidepressant treatment and ours on which bacteria were positively or negatively affected by antidepressant treatment. Other studies of gut microbiota in patients with MDD have observed altered bacteria-associated enzyme-linked genes coding for tryptophan biosynthesis and metabolism, as well as loss of tryptophan metabolites [34, 110]. As mentioned earlier, the genus *Desulfovibrio* is involved in neurotransmitter regulation, and a study has found it is specifically involved in tryptophan metabolism [111]. Therefore, increased *Desulfovibrio* after twelve weeks of antidepressant treatment may indicate that *Desulfovibrio* enhances the tryptophan availability, giving rise to higher serotonin availability. As most of our patients with MDD received selective serotonin reuptake inhibitors as antidepressant treatment, the combined effects of the treatment with increased *Desulfovibrio* may be associated with the decreased MDI scores. This is supported by a study that found that acute tryptophan depletion limits the antidepressant effect of serotonin reuptake inhibitors such as fluoxetine [112]. Furthermore, the observed increased relative abundance of *Lactobacillus* in MDD after treatment may result in elevated production of SCFAs [113], metabolites suggested to have antidepressant properties as they regulate tryptophan production [19]. One of these properties is also directly linked to serotonin, as SCFAs can induce serotonin production by intestinal enterochromaffin cells [25]. We furthermore found that several taxa changed in relative abundance in the nonMDD group, which is interpreted as naturally occurring gut microbiota fluctuations. These changes were, however, not as profound as changes observed in the MDD group.

We are unable to conclude that MDD and response to treatment is linked to bacterial variations in the gut microbiota. The patient group who received pharmaceutical intervention was relatively small (*n* = 11). It is therefore difficult to discern if the antidepressant treatment has robustly affected the gut microbial composition, especially since the patients were not administered the same type or class of antidepressants. Another reason for alterations in the gut microbiota of patients with MDD during the twelve weeks may have been an altered diet. Many antidepressant therapeutics have a common side effect, which is altered appetite [114]. Although patients did not report pronounced changes in caloric intake or appetite, it is well-known that self-report biases include both recall and social desirability bias, which include questions regarding dietary intake [115]. Patients may therefore, intentionally, or unintentionally, have under- or overestimated dietary intake, which can lead to alterations in the gut microbiota [74–76]. Overall, we cannot from this study deduce if antidepressant treatment can manipulate the gut microbiota of patients with MDD leading to increased treatment response.

### Limitations and strengths

The limitations of this study are the relatively low sample size due to difficulties in recruitment and a high attrition rate throughout the study. Patients were not administered the same types or classes of medicine, and in combination with the small sample size, it is therefore difficult to associate bacterial alterations with a specific type of antidepressant. The questionnaire concerning dietary habits did not track caloric intake, and patients may therefore have altered their dietary intake during the study, leading to some of the observed bacterial alterations. Due to 16 S rRNA amplicon sequencing being the method of bacteria identification, it was not possible to identify taxa on species or strain level.

This is the first study, to our knowledge, that assesses changes in gut microbiota of patients with MDD during antidepressant treatment and/or psychotherapy. A further strength of this study is the homogeneity of our MDD and nonMDD groups, as this limits biases induced by age, diet and comorbid disorders. Additionally, our patient cohort was treatment-naive at baseline, which has only been examined in one previous study [109].

## Conclusion

Although there were no significant differences in α- or β-diversity at baseline, or from baseline to twelve weeks, individual taxa were significantly different in relative abundance between MDD and nonMDD groups, and also over time. *Desulfovibrio, Gordonibacter* and *Rothia* were found to be different between the MDD and nonMDD groups at baseline, and furthermore changed in relative abundance during the antidepressant treatment and/or psychotherapy, indicating an association between these bacterial taxa and depression. Furthermore, the MDD group was found to have a predominantly pro-inflammatory profile compared to the nonMDD group at baseline, including associations between MDI and Bray-Curtis dissimilarity, as well as several immune biomarkers. Combined, these results indicate that there is a significantly different gut microbiota composition in patients with MDD, and that changes in these bacterial taxa are associated with both antidepressant treatment and decreased MDI score.

### Electronic supplementary material

Below is the link to the electronic supplementary material.

**Supplementary Material 1:** Full list of in- and exclusion criteria for both patients with MDD and non-depressed controls

**Supplementary Material 2: Supplementary Fig. 1.** Quality of sequencing. (**A**) Reads following sequencing for each group. (**B**) Rarefaction curve for each sample within each group

**Supplementary Material 3: Supplementary Fig. 2.** α-diversity measures representing comparisons between MDD and nonMDD at baseline, four weeks and twelve weeks follow-up

**Supplementary Material 4: Supplementary Fig. 3.** β-diversity measures representing comparisons between MDD and nonMDD at baseline, four weeks and twelve weeks follow-up

**Supplementary Material 5: Supplementary Fig. 4.** Heatmap representing the 25 most abundant species

**Supplementary Material 6: Supplementary Fig. 5.** Barplot representing the most abundant phyla

**Supplementary Material 7: Supplementary Fig. 6.** Longitudinal variations in immune markers observed to be significantly associated with the MDD group compared to the nonMDD group in Figure 5

**Supplementary Material 8:** Supplementary figure legends

## Data Availability

The datasets used and/or analyzed during the current study are available in the NCBI Sequence at https://www.ncbi.nlm.nih.gov/bioproject/PRJNA1054468.

## Abbreviations

ANCOM-BC: Analysis of Compositions of Microbiomes with Bias Correction
ASV: Amplicon sequence variants
BSS: Bristol Stool Scale
DSM: Diagnostic and Statistical Manual of Mental Disorders
ICD: International Classification of Diseases
LBP: Lipopolysaccharide-binding protein
LDA: Linear discriminant analysis
LPS: Lipopolysaccharide
MDD: Major depressive disorder
MDI: Major Depressive Inventory
PCR: Polymerase chain reaction
SCFA: Short chain-fatty acid
