# Supplementary material for: Differences in bacterial taxa between treatment-naive patients with major depressive disorder and non-affected controls may be related to a proinflammatory profile
Source: BMC Psychiatry. 2024 Jan 31;24:84. doi: 10.1186/s12888-024-05547-z (PMC10832199; doi:10.1186/s12888-024-05547-z)
Supplement: Supplementary file 1 — Supplementary Material 1: Full list of in- and exclusion criteria for both patients with MDD and non-depressed controls [file 12888_2024_5547_MOESM1_ESM.docx]

| Patients with major depressive disorder (MDD) | | |
| --- | --- | --- |
| *Inclusion criteria* | *Exclusion criteria* | |
| - Age 18-24 years, both years included. - Depressive episode as defined by ICD-10 diagnoses: F32.0, F32.1, F32.2, F.32.3, F.33.0, F.33.1, F.33.2, F.33.3 as determined by or under the supervision of a psychiatrist. | - Prior diagnosis with and/or pharmacological treatment of depression. - Infectious diseases within the last month prior to inclusion in the study requiring prescription medicine. - Any infection one month prior to the study, or during collection of samples as reported by the patient in the journal provided them. - Mental, behavioural, congenital, neurodevelopmental and neurological diseases that might affect cognition and/or central neurology (including, but not limited to organic mental disorders, eating disorders, personality disorders, intellectual disabilities, head trauma, extrapyramidal disorders, demyelinating diseases, or cerebral palsy) - Diseases of the digestive system as judged by investigator (including, but not limited to IBD and IBS). - Endocrine diseases implicated to be associated with gut dysbiosis, as judged by investigator. - Infectious or inflammatory joint disorders - Alcohol or drug abuse as judged by psychiatrist. - Current or previous pregnancy within the last year. - Use of antibiotics, probiotics, prebiotics or synbiotics a month prior to sampling. - Vegetarian / vegan / gluten-free diet | |
| Non-depressed individuals (nonMDD) | |  |
| *Inclusion criteria* | *Exclusion criteria* |  |
| - 18-30 years of age (both ages included) | - Current or previous diagnosis of depression - Prior treatment of depression - Infectious diseases within the last month prior to inclusion in the study requiring prescription medicine. - Any infection one month prior to inclusion, or during collection of samples as reported by the patient in the journal provided them. - Mental, behavioural, congenital, neurodevelopmental and neurological diseases that might affect cognition and/or central neurology (including, but not limited to organic mental disorders, eating disorders, personality disorders, intellectual disabilities, head trauma, extrapyramidal disorders, demyelinating diseases, or cerebral palsy) - Diseases of the digestive system as judged by investigator (including, but not limited to IBD and IBS). - Endocrinological diseases implicated to be associated with gut dysbiosis, as judged by investigator (including, but not limited to type 1 and type 2 diabetes, thyroid diseases and hormonal imbalances) - Infectious or inflammatory joint disorders - Alcohol or drug abuse as judged by psychiatrist. - Current or previous pregnancy within the last year. - Use of antibiotics, probiotics, prebiotics or synbiotics a month prior to sampling. - Vegetarian / vegan / gluten-free diet |  |

***Supplementary material 1* – Full list of in- and exclusion criteria for both patients with MDD and non-depressed controls.** IBD: Inflammatory bowel disorders. IBS: Irritable bowel syndrome.
