# Supplementary figures and images for: Differences in bacterial taxa between treatment-naive patients with major depressive disorder and non-affected controls may be related to a proinflammatory profile
Source: BMC Psychiatry. 2024 Jan 31;24:84. doi: 10.1186/s12888-024-05547-z (PMC10832199; doi:10.1186/s12888-024-05547-z)

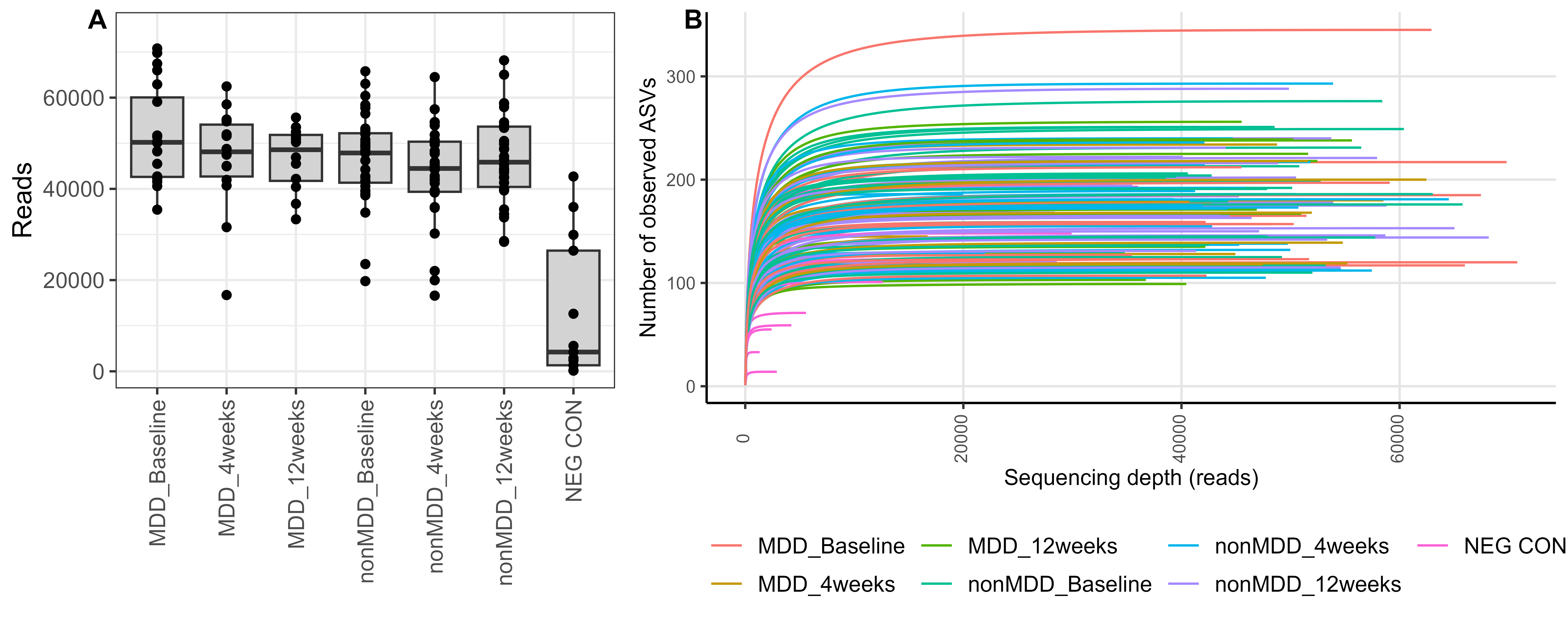

Supplement: Supplementary file 2 — Supplementary Material 2: Supplementary Fig. 1. Quality of sequencing. (A) Reads following sequencing for each group. (B) Rarefaction curve for each sample within each group [file 12888_2024_5547_MOESM2_ESM.png]

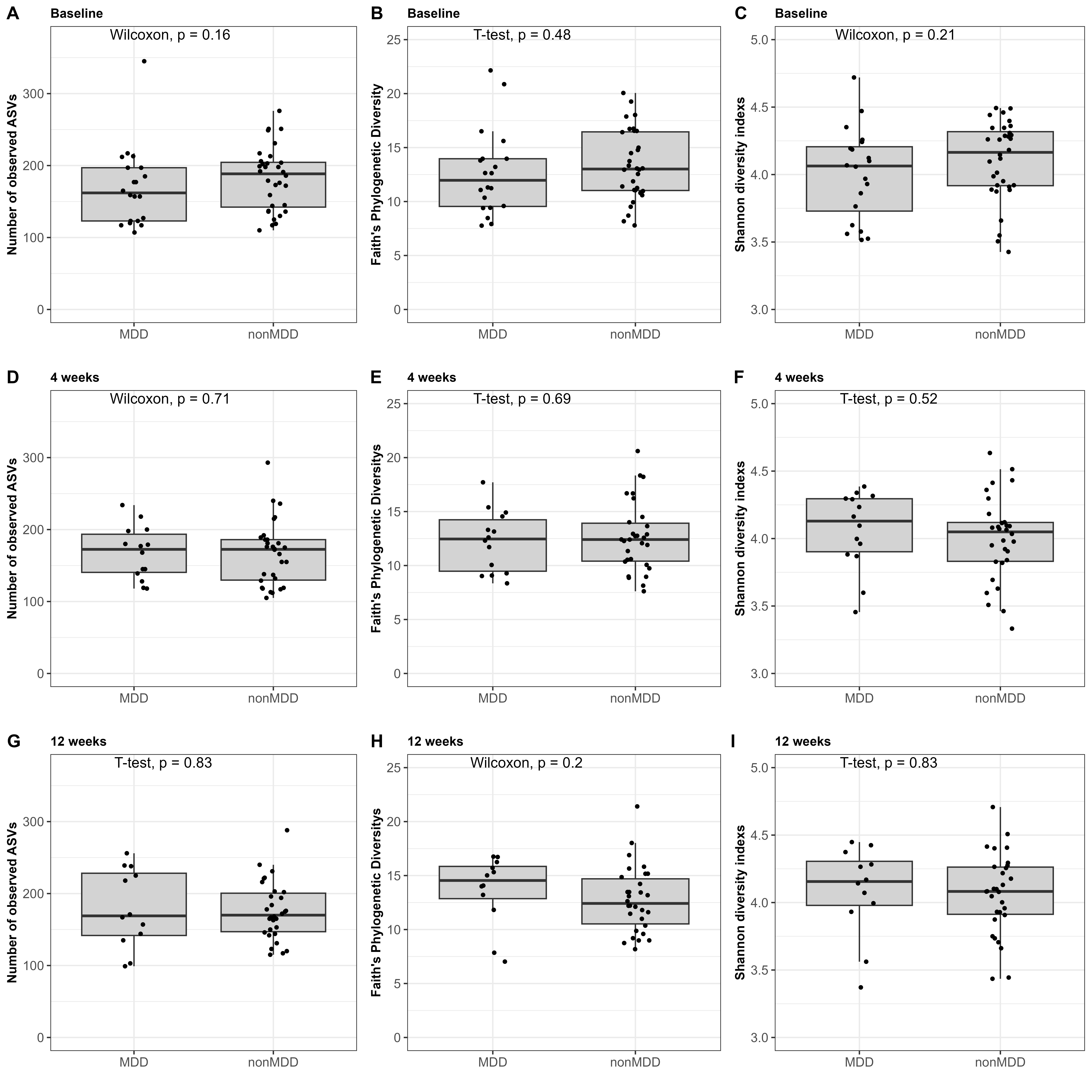

Supplement: Supplementary file 3 — Supplementary Material 3: Supplementary Fig. 2. α-diversity measures representing comparisons between MDD and nonMDD at baseline, four weeks and twelve weeks follow-up [file 12888_2024_5547_MOESM3_ESM.png]

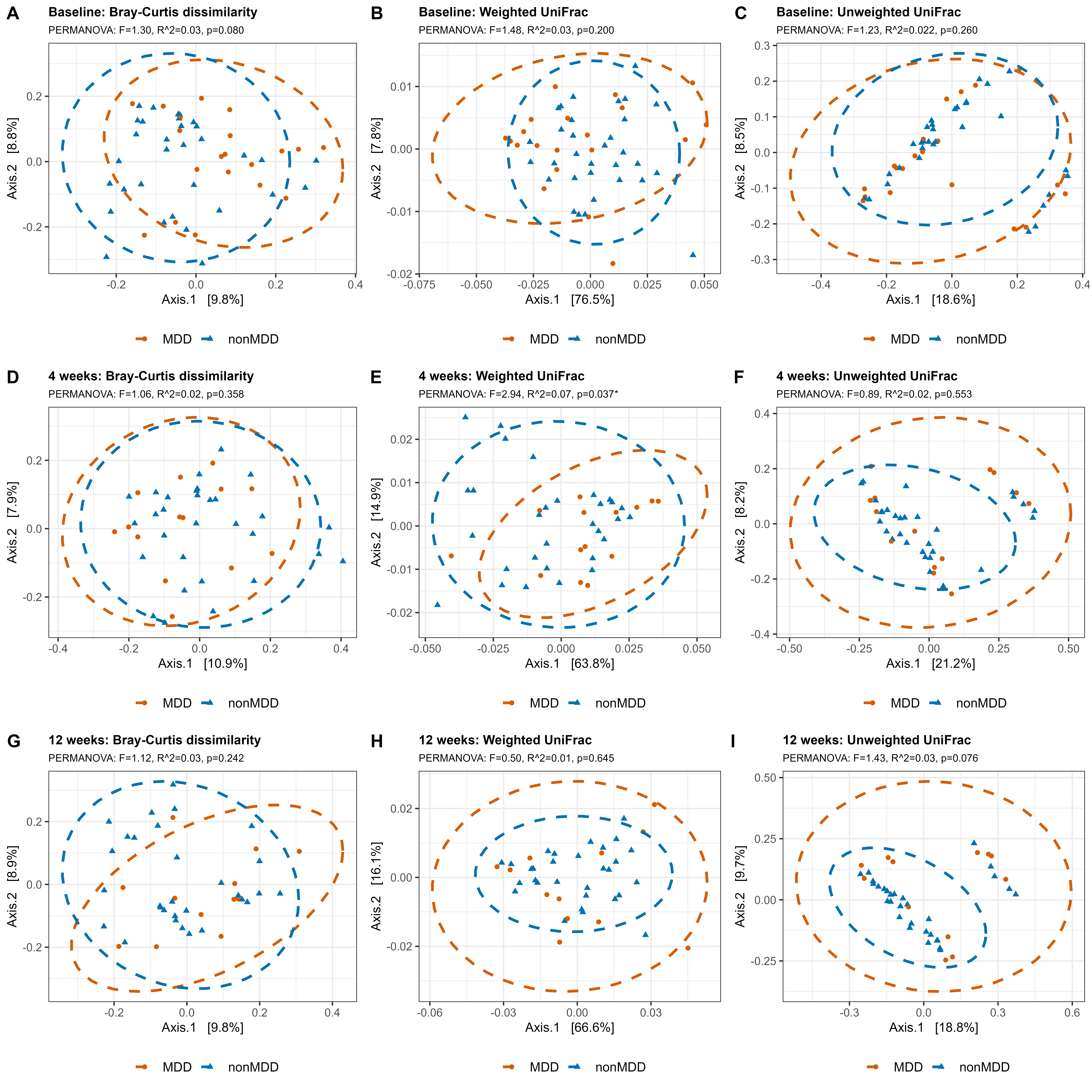

Supplement: Supplementary file 4 — Supplementary Material 4: Supplementary Fig. 3. β-diversity measures representing comparisons between MDD and nonMDD at baseline, four weeks and twelve weeks follow-up [file 12888_2024_5547_MOESM4_ESM.png]

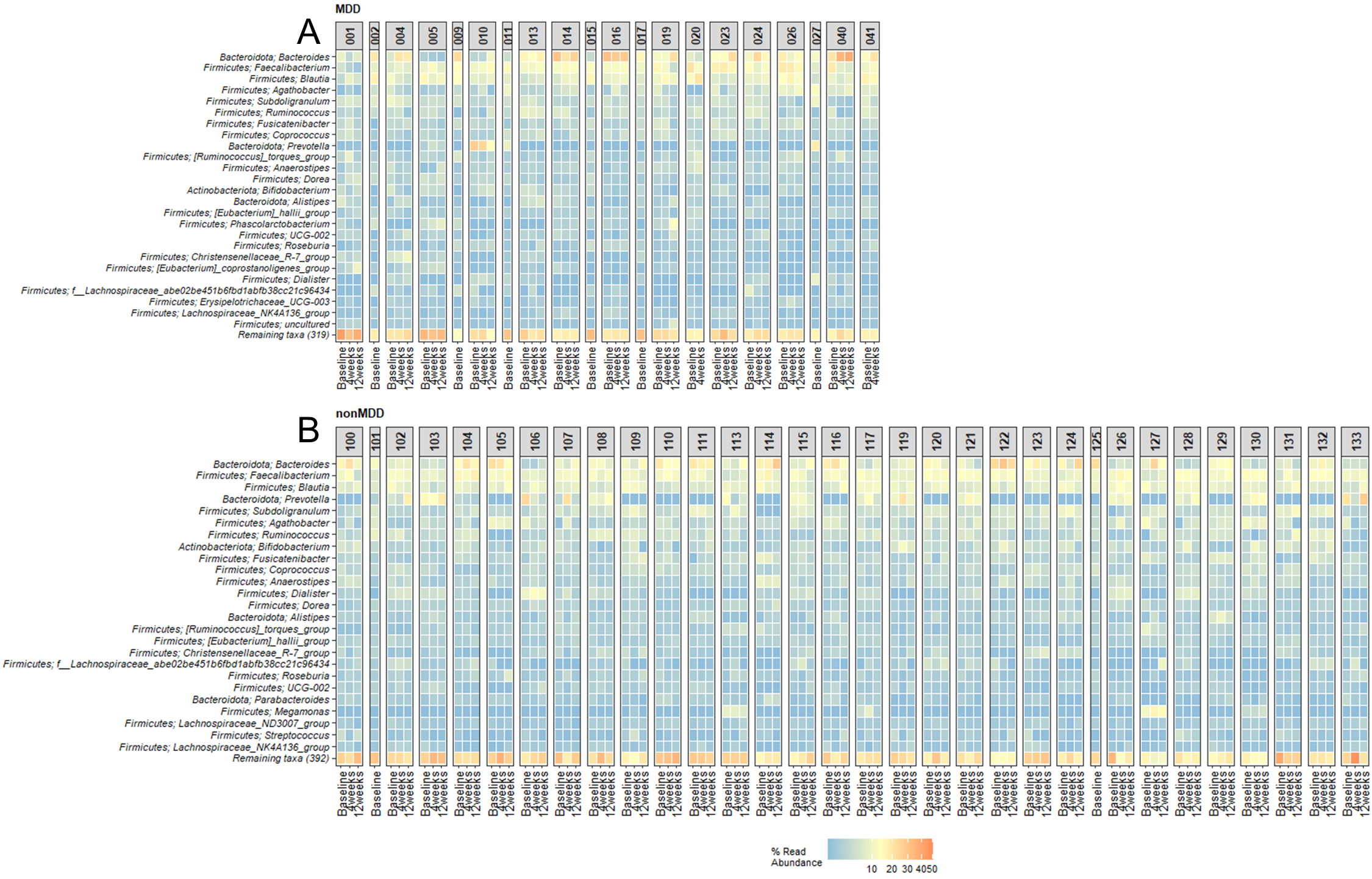

Supplement: Supplementary file 5 — Supplementary Material 5: Supplementary Fig. 4. Heatmap representing the 25 most abundant species [file 12888_2024_5547_MOESM5_ESM.png]

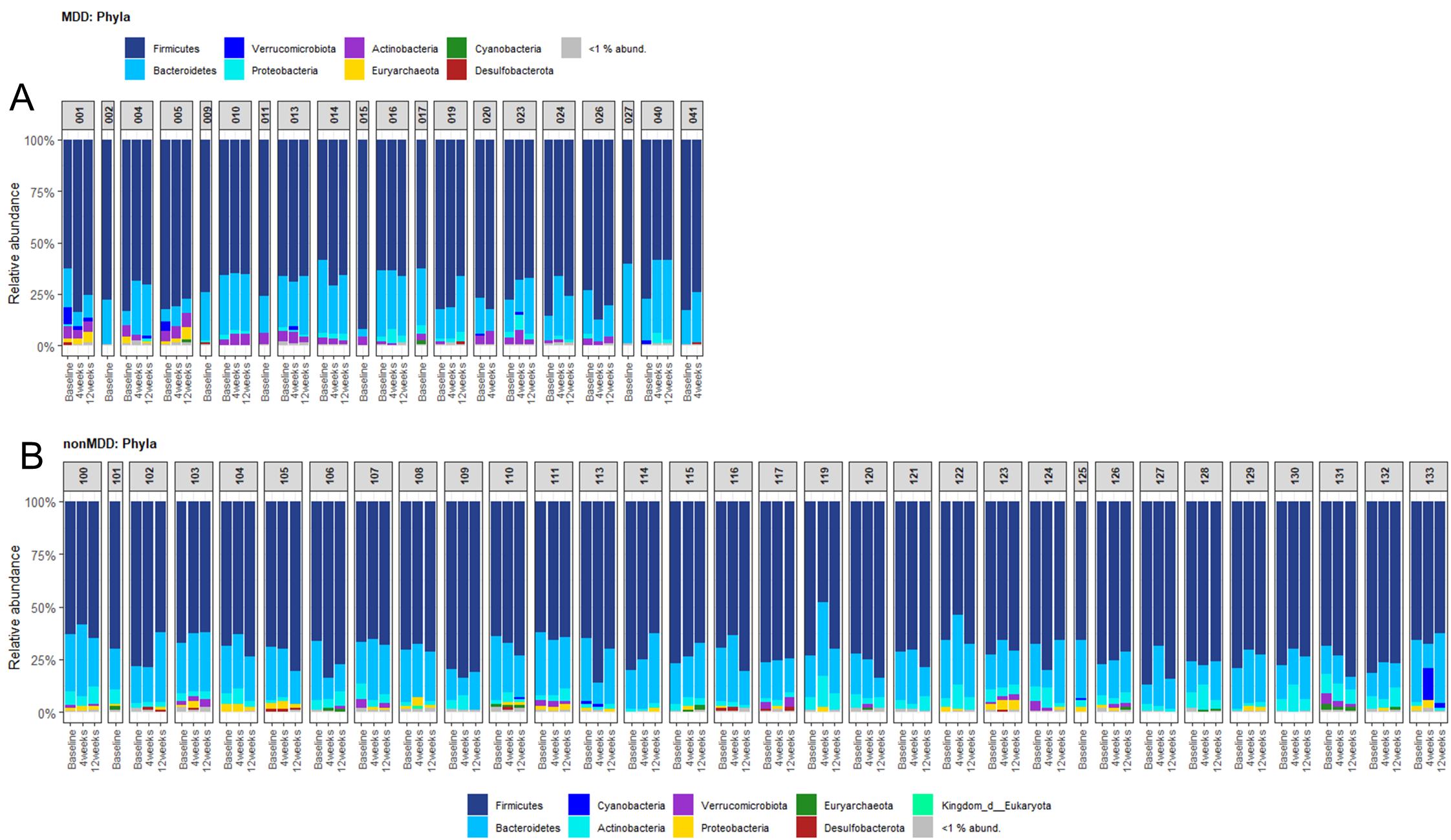

Supplement: Supplementary file 6 — Supplementary Material 6: Supplementary Fig. 5. Barplot representing the most abundant phyla [file 12888_2024_5547_MOESM6_ESM.png]

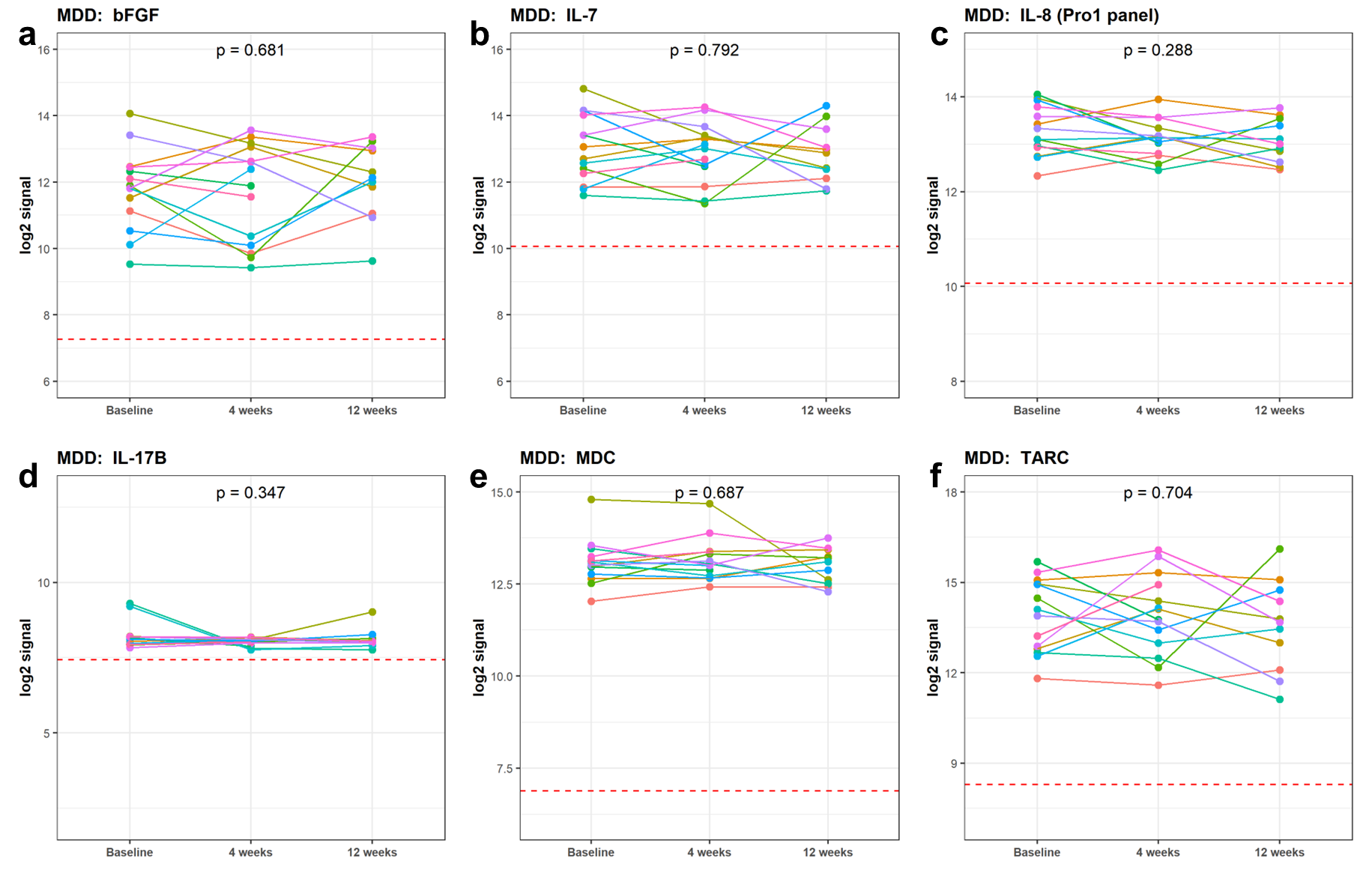

Supplement: Supplementary file 7 — Supplementary Material 7: Supplementary Fig. 6. Longitudinal variations in immune markers observed to be significantly associated with the MDD group compared to the nonMDD group in Figure 5 [file 12888_2024_5547_MOESM7_ESM.png]
