# Supplementary material for: Differences in bacterial taxa between treatment-naive patients with major depressive disorder and non-affected controls may be related to a proinflammatory profile
Source: BMC Psychiatry. 2024 Jan 31;24:84. doi: 10.1186/s12888-024-05547-z (PMC10832199; doi:10.1186/s12888-024-05547-z)
Supplement: Supplementary file 8 — Supplementary Material 8: Supplementary figure legends [file 12888_2024_5547_MOESM8_ESM.docx]

**Supplementary figure legends**

*Supplementary Figure 1* – Quality of sequencing. A) Reads following sequencing for each group. B) Rarefaction curve for each sample within each group.

*Supplementary Figure 2* – α-diversity measures representing comparisons between MDD and nonMDD at baseline, four weeks and twelve weeks follow-up. Diversity indices measured as number of observed ASVs, Faith´s phylogenetic index and Shannons diversity between MDD and nonMDD at baseline (A, B, and C), four weeks (D, E and F) and twelve weeks (G, H and I).

*Supplementary Figure 3* – β-diversity measures representing comparisons between MDD and nonMDD at baseline, four weeks and twelve weeks follow-up. PCoA plots describing Bray-Curtis Dissimilarity, Weighted UniFrac and Unweighted UniFrac at baseline (A, B and C), four weeks (D, E and F) and twelve weeks (G, H, and I).

*Supplementary Figure 4* – Heatmap representing the 25 most abundant species. The 25 most abundant species in the MDD group (A) at baseline, 4 weeks and 12 weeks follow up, and in the nonMDD group (B) at baseline, 4 weeks and 12 weeks follow up.

*Supplementary Figure 5* – Barplot representing the most abundant phyla. The most abundant phyla in the MDD group (A) at baseline, 4 weeks and 12 weeks follow up, and in the nonMDD group (B) at baseline, 4 weeks and 12 weeks follow up.

***Supplementary Figure 6 –* Longitudinal variations in immune markers observed to be significantly associated with the MDD group compared to the nonMDD group in *Figure 5***
